# Changes in Cyanobacterial Phytoplankton Communities in Lake-Water Mesocosms Treated with Either Glucose or Hydrogen Peroxide

**DOI:** 10.3390/microorganisms12091925

**Published:** 2024-09-22

**Authors:** David Linz, Charlyn G. Partridge, Michael C. Hassett, Nathan Sienkiewicz, Katie Tyrrell, Aimèe Henderson, Renee Tardani, Jingrang Lu, Alan D. Steinman, Stephen Vesper

**Affiliations:** 1 Oak Ridge Institute for Science and Education, Oak Ridge, TN 37831, USA; linz.david@epa.gov; 2 Annis Water Resources Institute, Graduate School, Grand Valley State University, Allendale, MI 49441, USA; partridc@gvsu.edu (C.G.P.); hassetmi@gvsu.edu (M.C.H.); tyrrellk@gvsu.edu (K.T.); tardanir@mail.gvsu.edu (R.T.); steinmaa@gvsu.edu (A.D.S.); 3 United States Environmental Protection Agency, Cincinnati, OH 45268, USA; sienkiewicz.nathan@epa.gov (N.S.); henderson.aimee@epa.gov (A.H.); lu.jingrang@epa.gov (J.L.)

**Keywords:** cyanobacteria, glucose, hydrogen peroxide, mesocosm, freshwater

## Abstract

When cyanobacterial phytoplankton form harmful cyanobacterial blooms (HCBs), the toxins they produce threaten freshwater ecosystems. Hydrogen peroxide is often used to control HCBs, but it is broadly toxic and dangerous to handle. Previously, we demonstrated that glucose addition to lake water could suppress the abundance of cyanobacteria. In this study, glucose was compared to hydrogen peroxide for the treatment of cyanobacterial phytoplankton communities. The six-week study was conducted in the large mesocosms facility at Grand Valley State University’s Annis Water Resources Institute in Michigan. To 1000 L of Muskegon Lake water, glucose was added at either 150 mg or 30 mg glucose/L. Hydrogen peroxide was added at 3 mg/L to two 1000 L mesocosms. And two mesocosms were left untreated as controls. Triplicate 100 mL samples were collected weekly from each mesocosm, which were then filtered and frozen at −80 °C for 16S rRNA amplicon sequencing. The 16S rRNA amplicon sequencing results revealed that hydrogen peroxide treatment quickly reduced the relative abundance of the cyanobacteria compared to the control mesocosms, but the cyanobacteria population returned over the course of the 6-week study. On the other hand, both glucose concentrations caused a rapid proliferation of multiple low abundance proteobacterial and bacteroidotal taxa resulting in notable increases in taxonomic richness over the duration of the study and reducing the relative abundance of cyanobacteria. Although hydrogen peroxide quickly suppressed the cyanobacteria, the population later returned to near starting levels. The glucose suppressed the cyanobacterial phytoplankton apparently by promoting competitive heterotrophic bacteria.

## 1. Introduction

Phytoplankton are the major primary producers in aquatic environments. Temperature, light variations, and the annual cycle of nutrient availability drive phytoplankton community dynamics [1]. The combination of eukaryotic algae, cyanobacteria, and heterotrophic bacteria forms the phycosphere [2]. The dynamics of the phycosphere are determined by the size and depth of a waterbody, its hydrology, nutrient concentrations and many other factors [3,4,5]. These natural cycles are now being disrupted by anthropogenic factors leading to harmful cyanobacterial blooms (HCBs), also called harmful algal blooms (HABs).

Blooms of cyanobacteria are occurring more frequently in freshwater [6]. Although cyanobacteria are a natural component of the freshwater ecosystem, the addition of excess nutrients in combination with climate change appears to be promoting HCBs [7]. The long-term solution to these blooms is to eliminate the excess nutrients and mitigate climate change. In the short term, methods are needed to treat the water to prevent blooms. Hydrogen peroxide is currently used to suppress cyanobacterial growth in freshwater [8,9]. As a possible alternative to hydrogen peroxide treatment, glucose was tested and found to suppress cyanobacteria in small mesocosms containing water from an Ohio lake [10,11]. In this study, large mesocosms filled with 1000 L of water from Muskegon Lake in Michigan (USA) were used to compare changes in the cyanobacterial community, after glucose or hydrogen peroxide treatments.

## 2. Material and Methods

### 2.1. Muskegon Lake and the AWRI Research Facility 

Muskegon Lake is a mesotrophic, drowned river-mouth lake located on the west coast of Michigan’s lower peninsula. The lake has a long history of industrial activity on its shoreline, resulting in its designation as a Great Lakes Area of Concern [12]. The lake has a maximum depth of 24 m, a surface area of 16.8 km^2^, and an average hydraulic residence time of ~23 days; however, this can range from 14 to 70 days depending on the season [13,14]. The mesocosm facility at Grand Valley State University’s Annis Water Resources Institute (AWRI) on Muskegon Lake was utilized for this study.

The AWRI facility pumps Muskegon Lake water through a series of in-line filters (300 µm pore size) to a head tank, where it is gravity fed into each mesocosm tank. The 1325 L fiberglass mesocosm tanks are illuminated with 1000 W metal halide lamps suspended above each tank to provide full-spectrum photosynthetically active radiation (~300 µmol m^2^ s^1^) with a photoperiod of 16 h light (L)/8 h dark (D). Ambient light (mean = 55 µmol m^2^ s^1^) was also present for a 14 L:10 D period. 

### 2.2. Experimental Design

On 14 August 2023, 1000 L of filtered Muskegon Lake water was added to six mesocosms. The water in the mesocosms was allowed to equilibrate overnight. On 15 August, two mesocosms were each treated with hydrogen peroxide at 3 mg/L and designated hydrogen peroxide mesocosms A and B. In addition, two mesocosms were treated with glucose, either at 150 mg/L, designated the glucose-high mesocosm, or at 30 mg/L, designated the glucose-low mesocosm. As controls, two of the mesocosms were left untreated and designated control mesocosms A and B. The water in each tank was hand-stirred daily with individual wooden dowels assigned to each mesocosm. 

### 2.3. Mesocosm Sampling and Amplicon Sequencing

During the six-week experiment, triplicate 100 mL samples were obtained weekly from each mesocosm and filtered through Durapore polyvinylidene fluoride (PVDF) filters, with 0.45 μm pore size (MilliPore, Foster City, CA, USA). Each filter was inserted into a 2 mL Matrix Lysis-A tube (MP Biomedicals, LLC, Santa Ana, CA, USA) and 600 μL RLT plus buffer was added to each tube (QIAGEN, Valencia, CA, USA). The tubes were labeled and frozen at −80 °C until they were processed for high-throughput sequencing, as previously described [11]. 

Sequencing and library preparation were performed to target the 16S rRNA v3-4 region. The methods used were described by Bagley et al. [15] with the following modifications. The first round PCR was performed with 22 µL Accuprime pfx supermix (Thermo-Fisher, Waltham, MA, USA), 0.5 µL of each primer, and 2 µL of DNA. After gel confirmation of amplification products, PCR products were cleaned with 19 µL of AMPure XP beads (Beckman Coulter, Brea, CA, USA), added to 23 µL of the PCR products, and eluted in 35 µL of 10 mM Tris pH 8.5. PCR products were normalized to 5 ng/µL. An index PCR was performed using 22 µL Accuprime pfx supermix, 0.5 µL of 10 µM of each index primer, and the product was cleaned using 27.5 µL AMPure XP added to 25 µL of PCR product and eluted with 35 µL of 10 mM Tris pH 8.5. Samples were normalized to a concentration of 4 nM and 5 µL of each were combined to make the final library. The pooled library was sequenced using a 600 cycle V3 MiSeq sequencing kit (# MS-102-3003, Illumina, San Diego, CA, USA) according to manufacturer’s protocol, using 2 × 300 paired-end sequencing. 

### 2.4. Amplicon Processing

Raw demultiplexed reads, with adapters removed, were processed using the software suite QIIME 2 2021.4.0 [16]. Raw sequence data were quality filtered, denoised, and cleared of chimeras with DADA2 (via q2-dada2) [17]. Taxonomy was assigned to amplicon sequence variants (ASVs) based on the Silva 138 SSU reference using the q2-feature-classifier [18] and the classify-sklearn naive Bayes taxonomy classifier [19,20]. Qiime2 artifacts were then moved to R v4.1.2 using the qiime2R package for further analysis [21]. 

### 2.5. Sequence Data Analysis

Analysis of the final sequence dataset was performed in R v4.1.2 [22] using the packages phyloseq (v1.38.0) [23], vegan (v2.5.7) [24] and visualized using ggplot2 (v3.3.5) [25]. Samples were initially pruned of non-bacterial, unidentified taxa, and ASVs were assigned as “chloroplast”. Replicates for each sample were initially examined and, after determining consistency among replicates, merged with phyloseq’s “merge_samples” function for subsequent analysis. For certain components of our analysis, low-abundance taxa (<5%) were removed from the dataset. Bray–Curtis between-sample distances were computed at the phylum level. Distance matrices were then used to cluster samples using non-metric multidimensional scaling (NMDS). The envfit function was used to understand the phyla most significantly correlating with our ordination. Alpha diversity (observed taxa) was assessed at the species level for all bacteria and for the respective phyla of interest.

## 3. Results 

**Figure 1:**
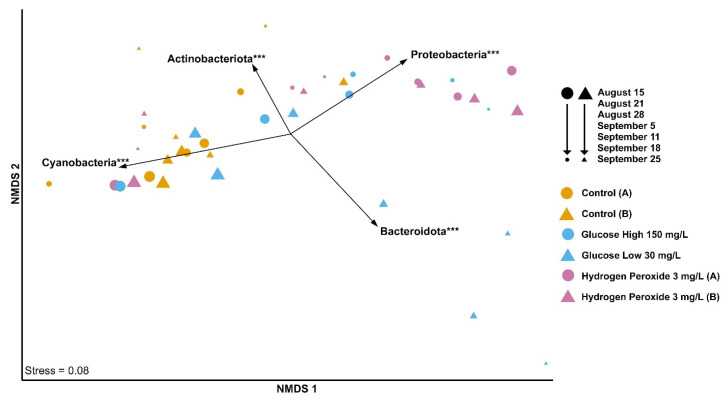
Non-metric multidimensional scaling (NMDS) plot of Bray–Curtis distances for the six mesocosms: control A = yellow circle, control B = yellow triangle; glucose-high = blue circle, glucose-low = blue triangle; and hydrogen peroxide A = purple circle, hydrogen peroxide B = purple triangle. Date progression is represented by decreasing shape size. Phylum abundance was fitted as vectors to the NMDS using envfit. Only those with the most significant associations to NMDS clustering are shown. Arrow direction indicates increasing abundance and arrow length represents strength of correlation. *** = *p* < 0.001.

Based on the 16S rRNA amplicon sequencing, the general bacterial community composition across mesocosms was plotted using ordination with non-metric multidimensional scaling (NMDS) and the most significant phyla were projected as vectors (Figure 1). Initially, all mesocosm samples were generally consistent across mesocosms and enriched for the phylum Cyanobacteria (r^2^ = 0.91, *p* < 0.001). As the experiment proceeded, the hydrogen peroxide mesocosms and the glucose-high mesocosm developed increasing levels of proteobacteria (r^2^ = 0.83, *p* < 0.001), but the glucose-low mesocosm developed more bacteroidota (r^2^ = 0.88, *p* < 0.001) (Figure 1). 

**Figure 2:**
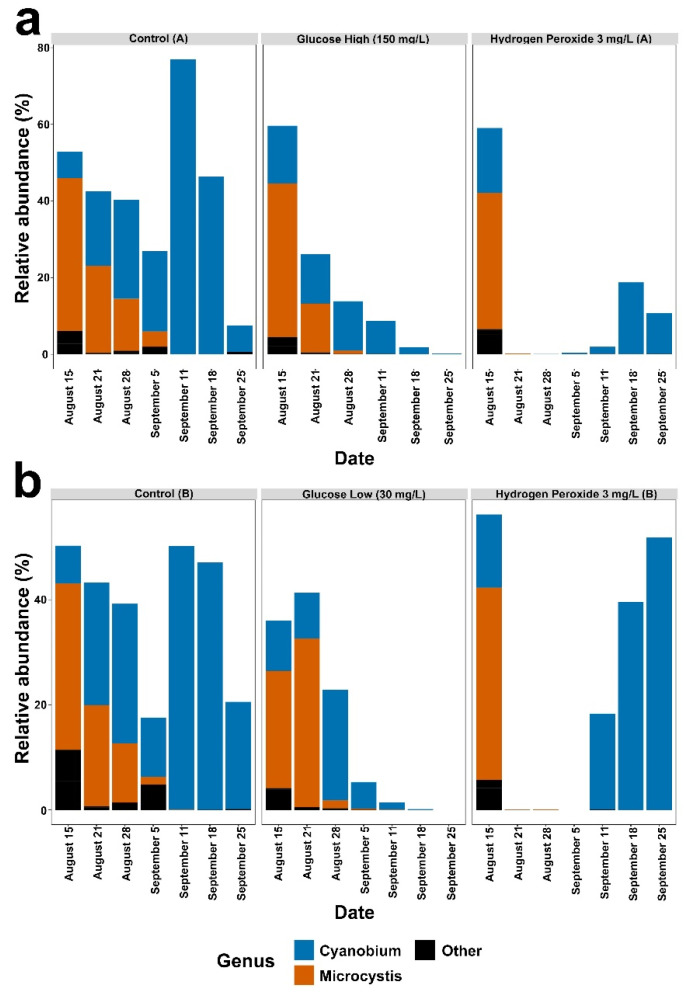
Results for Control (A), Glucose (A) and Hydrogen Peroxide (A) mesocosms are shown in panel (**a**). The results for Control (B), Glucose (B) and Hydrogen Peroxide (B) mesocosms are shown in panel (**b**). The relative abundance of Cyanobacteria genera occurring above 5% (compared to all bacteria) in the six mesocosms (control A or B, glucose-high or glucose-low, and hydrogen peroxide A or B) at each time point sampled and organized by date (x-axis). Genera below 5% relative abundance are grouped as ‘Other’ (black bars).

**Figure 3:**
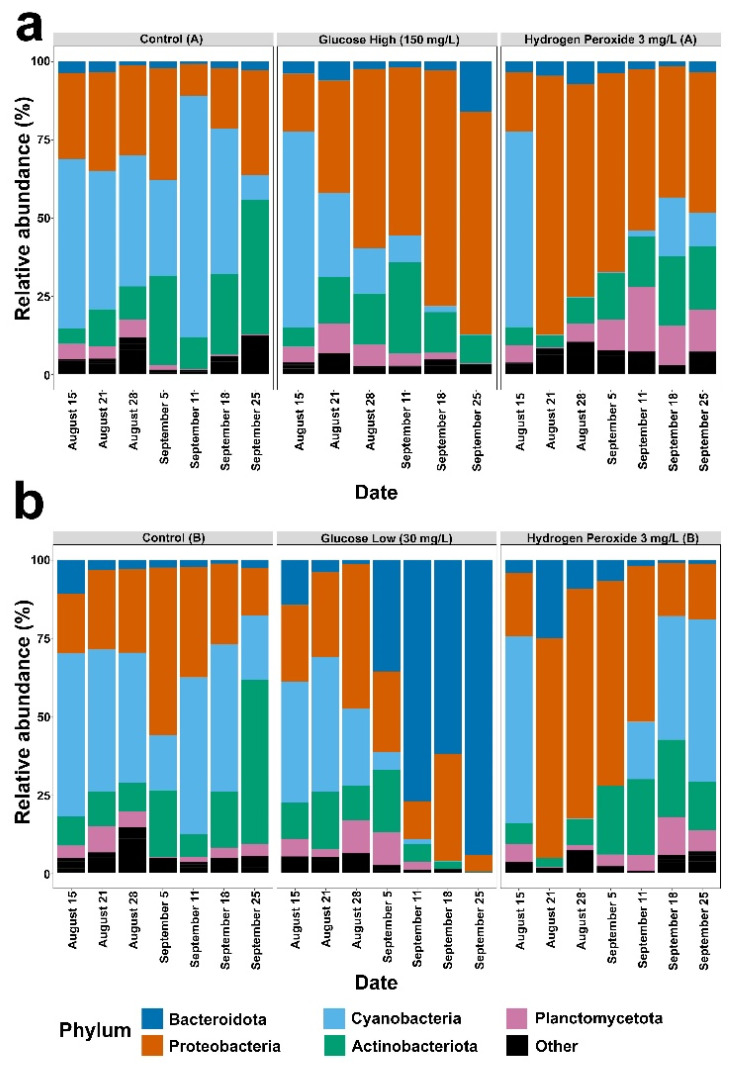
Results for Control (A), Glucose (A) and Hydrogen Peroxide (A) mesocosms are shown in panel (**a**). The results for Control (B), Glucose (B) and Hydrogen Peroxide (B) mesocosms are shown in panel (**b**). Relative abundance of bacterial phyla occurring above 10% (compared to all bacteria) in the six mesocosms (control A or B, glucose-high or glucose-low, and hydrogen peroxide A or B) at each time point sampled and organized by date (x-axis). Phyla below 10% relative abundance are grouped as ‘Other’ (black bars).

The genera of cyanobacteria detected in the mesocosms during the experiment were *Cyanobium* (R. Rippka & G. Cohen-Bazire) and *Microcystis* (Lemmermann) (Figure 2). At the phylum level, control mesocosms primarily consisted of cyanobacteria, but cyanobacteria levels gradually fell and Actinobacteriota levels began to increase (Figure 3, Control A and B). In the hydrogen peroxide treated mesocosms, cyanobacteria were rapidly reduced after about one week, allowing proteobacteria to become the dominant phylum (Figure 3, hydrogen peroxide A and B). Over time, the cyanobacteria gradually increased in relative abundance in the hydrogen peroxide mesocosms to levels comparable to the starting conditions. In both the glucose-high and glucose-low mesocosms, the relative abundance of cyanobacteria diminished (Figure 3) and proteobacteria became the dominant phylum in the glucose-high treated mesocosm (Figure 3a), while the glucose-low mesocosm showed an increase in the relative abundance of bacteroidota (Figure 3b).

**Figure 4:**
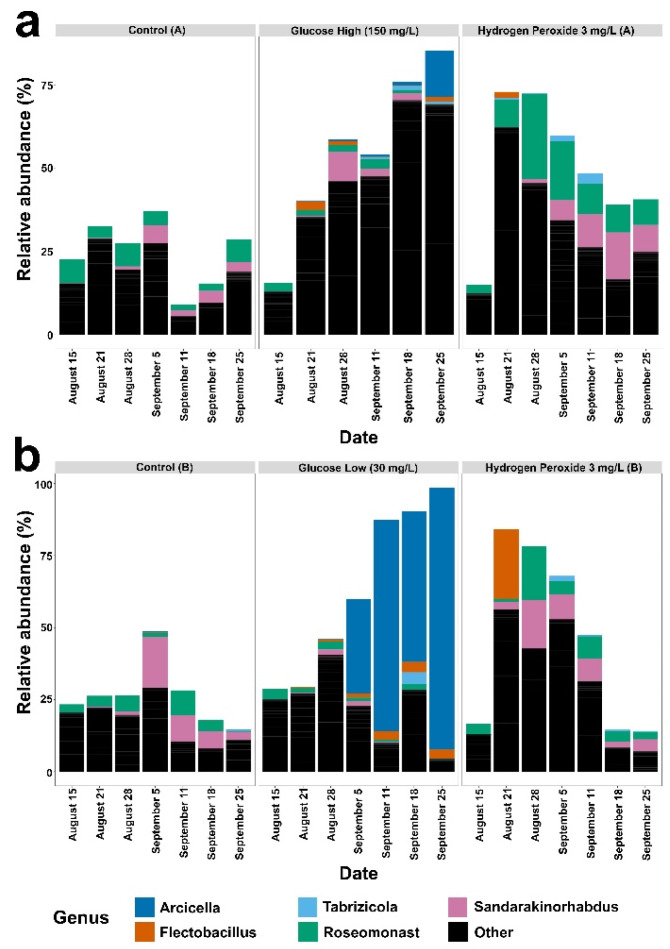
Results for Control (A), Glucose (A) and Hydrogen Peroxide (A) mesocosms are shown in panel (**a**). The results for Control (B), Glucose (B) and Hydrogen Peroxide (B) mesocosms are shown in panel (**b**). Relative abundance of all genera of proteobacteria and bacteroidota occurring above 10% (compared to all bacteria) in the six mesocosms (control A or B, glucose-high or glucose-low, and hydrogen peroxide A or B) at each time point sampled and organized by date (x-axis). Genera below 10% relative abundance are grouped as ‘Other’ (black bars).

**Figure 5:**
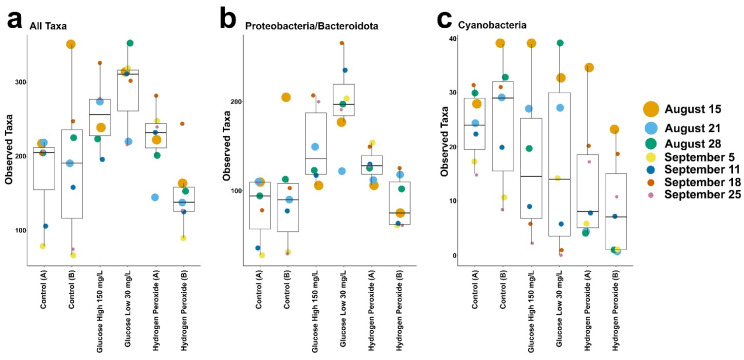
Raw observed taxa in the six mesocosms (control A or B, glucose-high or glucose-low, and hydrogen peroxide A or B) with date indicated by color and size (legend on right applies to all panels). (**a**) All taxa. (**b**) Proteobacteria/bacteroidota. (**c**) Cyanobacteria. Mesocosms are labeled on the x-axis. Note that the y-axis has differed scales for each panel.

Therefore, the relative abundance of genera in the proteobacteria and bacteroidota phylum were examined in each mesocosm. We found that the shift in proteobacteria and bacteroidota communities was primarily driven by diverse low-abundance genera (Figure 4—“Other”) and, in nearly all mesocosms, the identified genera from the proteobacteria or bacteroidota were below 10% relative abundance (Figure 4). As an exception to this observation, the glucose-low mesocosm showed a distinct increase in the relative abundance of the bacteroidota genus *Arcicella* (Nikitin) (Figure 4) compared to the glucose-high mesocosm. Both glucose treatments caused an increase in the overall number of observed taxa, i.e., community richness, compared to the control mesocosms and hydrogen peroxide treated mesocosms (Figure 5a). The proteobacteria/bacteroidota increased in the glucose treated mesocosms (Figure 5b) with a corresponding decrease in the observed number of cyanobacterial taxa (Figure 5c). 

## 4. Discussion

In the summer of 2023, *Microcystis* and *Cyanobium* were the dominant cyanobacteria in the mesocosm water. *Microcystis* and *Cyanobium* are common bloom-forming genera [26,27]. Microcystis is often a dominant taxon in Muskegon Lake [28]. Other components of the bacterioplankton community included the phyla proteobacteria (~25%), bacteroidota (~10%) and Actinobacteriota (~10%), which is consistent with other human-impacted freshwater lakes around the world [29,30]. 

Many physical, biological, and chemical treatments have been tested to control HCBs, but various concentrations of hydrogen peroxide are commonly utilized. In a laboratory study, hydrogen peroxide at 3 mg/L suppressed *Microcystis* [31]. In three lakes in the Netherlands, Piel et al. [32] used 2–5 mg/L hydrogen peroxide to control cyanobacteria. The treatment nearly eliminated the cyanobacteria, but new blooms developed within a few weeks. Because of hydrogen peroxide’s toxicity, rotifer populations strongly declined. Chen et al. [33] used 4 to 20 mg/L hydrogen, which resulted in an increase in eukaryotic algae and the dominant taxon of cyanobacteria changed from the nontoxic genus *Dactylococcopsis* to the toxic genus *Oscillatoria*. Huang et al. [34] demonstrated that high initial hydrogen peroxide concentrations resulted in an increase in pH, which increased hydrogen peroxide consumption. Lusty and Gobler [35] found that hydrogen peroxide reduced but did not fully eliminate cyanobacteria from eutrophic water bodies and suggested hydrogen peroxide may not be an ideal mitigation approach in high biomass ecosystems. In a recent meta-analysis of HCB treatment technologies, Anantapantula and Wilson [36] suggested that more research on existing and alternative treatments was needed.

Glucose has been proposed as a possible alternative to the use of hydrogen peroxide [10,11]. In this experiment, hydrogen peroxide acted faster than glucose in suppressing the cyanobacteria but maintained only a short efficacy. However, hydrogen peroxide had only minimal effects on the relative abundance of the accompanying bacterioplankton communities. On the other hand, glucose treatments took longer to be effective in suppressing the cyanobacteria. It appears that this delay is associated with the time for the proliferation of proteobacteria and bacteriodota, which likely outcompeted the cyanobacteria, causing their decrease in relative abundance later in the experiment.

We recognize the limitations of this large mesocosm study in replicating the dynamics of the lake itself with its variable light intensity, temperature changes, wind driven mixing, and the additional inputs of nutrients over time. For example, the contained nature of mesocosm water may be responsible for the gradual decline in cyanobacteria even in the control mesocosms. It remains to be seen how glucose additions to the lake itself would change the cyanobacterial community.

## 5. Conclusions

Glucose treatment should be tested further to determine if glucose additions can have any practical value in controlling HABS. 

## Data Availability

All data will be available at the NIH-PMC website. All sequence data have been deposited in the NCBI sequence read archive at accession number: PRJNA1114712.
